# A commentary on evidenced-based parenting programs: redressing misconceptions of the empirical support for Triple P

**DOI:** 10.1186/1741-7015-10-145

**Published:** 2012-11-22

**Authors:** Matthew R Sanders, John A Pickering, James N Kirby, Karen MT Turner, Alina Morawska, Trevor Mazzucchelli, Alan Ralph, Kate Sofronoff

**Affiliations:** 1 The University of Queensland, St Lucia, QLD 4072, Australia; 2 Curtin University, Perth, WA 6102, Australia

**Keywords:** Triple P, Public Health, Parenting, Evidence

## Abstract

A meta-analytic review of the Triple P-Positive Parenting program by Wilson *et al*., recently published in *BMC Medicine*, claimed to demonstrate that although Triple P is widely disseminated and adopted, the evidence attesting to the effectiveness of the program is not as convincing as it may appear. Although this review addresses the important issue of evaluation and reporting methods within evidence-based interventions, we contend that the Wilson *et al*. review contains a number of significant conceptual, methodological and interpretational inadequacies that render the key conclusions of their review problematic.

## Background

Wilson *et al. *[1] recently published a paper in this journal appraising the evidence underpinning the Triple P system of parenting interventions. Critical assessment of the literature supporting parenting programs is welcome, and the authors make several useful observations about how to enhance reporting standards and the value of clinical trial registration. The main conclusion in the paper was that despite evidence showing significant positive effects on maternal reports of child behavior (*ES *= .61), there are concerns about these effects due to investigator bias, inadequate reporting and conflicts of interest. The authors also concluded that there is no convincing evidence supporting the long-term or whole-of-population level benefits of Triple P. We argue that the strength of these conclusions is out of proportion to the evidence presented to support them, and detail our concerns with the authors' interpretation of their findings.

## Interpretation of findings

The conclusions reached by Wilson *et al. *cast doubt over the entire Triple P evidence-base. However, their conclusions hinge on a restrictive and narrow evaluation framework, primarily resulting in an inappropriate pooling across differing types of intervention studies, and exclusive focus on child-only outcome data. These limitations restrict the conclusions that can reasonably be drawn on the basis of their analysis.

Triple P is a blended model that incorporates both universal and indicated interventions for vulnerable families [2]. It is based on social learning theory and cognitive-behavioral principles, and is not a single intervention program. It is a tiered multilevel system of parenting support that has both preventive and treatment components and incorporates five levels of intensity and several delivery formats (for example, large group, small group, individual, self-directed, media and online interventions), with different variants and applications targeting different types of clinical problems, age groups and populations [2-4]. The evaluation of such a system of intervention is inevitably complex. By simply pooling studies, Wilson *et al. *fail to take into account differences in type and intensity of the interventions employed. The studies pooled included interventions of greatly varying intensity, modes of delivery and contact hours from self-help workbooks, and watching a brief television series, to intensive face-to-face behavioral family intervention. The random effects model employed was not sufficient to control for the differences across studies in terms of the type of intervention (for example, Stepping Stones, Indigenous, Workplace) and intervention intensity (for example, Level 1-5, Group, Individual, Self-Directed). An alternative approach would be to conduct subgroup analyses or moderator analyses examining these intervention differences. This approach would have provided a more accurate representation of the effects of the Triple P System of interventions.

A significant limitation of the Wilson *et al. *review is that it only reports on a narrow range of child outcomes. Importantly, there was no examination of any parent- or family-level outcomes, which are the primary targets of parenting interventions. Wilson *et al*.'s rationale for only focusing on child-based outcomes ignores the importance of parent- and family-level outcomes. The analysis ultimately reports a narrow index of child outcome data, using an inadequate pooled model of effects, which is insufficient to derive clear conclusions about the overall effectiveness of the Triple P system.

## Allegations of bias

Wilson *et al. *conclude that there is a strong risk of bias across Triple P research, stemming from high levels of authorial affiliation with Triple P, selective reporting and conflicts of interest. These claims of bias are both misleading and inaccurate.

### Authorial affiliation

Wilson *et al. *claim that most of the Triple P evidence has been 'authored by affiliates of the Triple P organization'. No evidence is cited to support this assertion and it is simply not true. Over 300 different authors across dozens of institutions in many countries worldwide have contributed to the Triple P evidence-base, including journal articles, books, book chapters and policy documents. Most are independent scholars with no formal affiliation to the University of Queensland or its licensed publisher, Triple P International. When the full evidence-base for Triple P is taken into account, there are 140 outcome studies, 43% of which have not involved a University of Queensland author. Exclusion of research published in languages other than English may have added to this misconception.

Developer-led research has had an important role in developing evidence-based parenting interventions, and is not necessarily any more biased than research conducted by independent evaluators when evaluators have known theoretical biases or preferences. Wilson *et al. *point out that outcome reporting bias is also an important consideration with respect to the interpretation of meta-analyses. As with any study, erroneous conclusions can be made when independent evaluations are implemented with poor fidelity, selectively report available evidence, exaggerate claims of independence, and have findings which are not replicated and are at variance with other studies. In their competing interests section, Wilson *et al. *state that one of the review's authors is a co-author of another parenting program, which is an important consideration in interpreting the findings of the review.

We also make the point that developer-led studies have been crucial to the field of evidence-based psychological and social interventions, and developers' involvement in the ongoing research and development process is highly desirable as it allows interventions to evolve, innovations to take place, and for the program developers to respond constructively to available evidence (including null findings) [5]. A hallmark of cognitive-behavioral interventions for parents has been the active, ongoing involvement of developers in the research and development process, and a preparedness of many developers to allow their interventions to evolve. This has encouraged a self-reflective, critical appraisal and gradual improvement of programs.

### Selective reporting

The multilevel nature of the Triple P system also influences the selection of outcome variables that are reported in different studies. Many studies included in the meta-analysis had a specific aim of examining the effects of Triple P on aspects of parenting and family functioning, including parental cognition, parental stress, parental depression, work-family conflict and couples' relationships. These variables speak to a broader range of potentially modifiable risk and protective factors in a child's family, and are critically important to determining children's developmental outcomes and the effectiveness of a parenting program. Wilson *et al. *have not taken into account the different aims of the various studies and the hypothesis-driven nature of the reporting of findings in individual studies included in their analysis.

### Conflict of interest

Triple P is not owned by its authors, but by The University of Queensland. Royalty payments from dissemination activities, principally the sale of books, are paid by the publisher (Triple P International) to the University of Queensland's technology transfer company (UniQuest), and distributed to the university's Faculty of Social and Behavioural Sciences, School of Psychology, Parenting and Family Support Centre and contributory authors in accordance with the university's intellectual property policy. None of the program authors own shares in Triple P International, the company licensed by the University of Queensland to disseminate the program worldwide. The Parenting and Family Support Centre has a formal policy of not disseminating any Triple P intervention that has not established an evidence-base sufficient to justify inclusion in the system. The research team has published trials showing minimal effects for specific parenting interventions which have not been disseminated [for example, [6,7]]. An international research network has been established to promote high quality developer-led and independent evaluations of Triple P.

## Contentious conclusions regarding the value of a public health approach to parenting support

Population-based approaches to parenting support are relatively new and represent a paradigm shift in the child maltreatment area on how to reduce the community prevalence of inadequate and abusive parenting. The Wilson *et al. *paper stressed the inadequacies of a public health approach to parenting support, highlighting the virtues of a traditional, more targeted approach to working with high-risk families. One of Wilson *et al*.'s main interpretations of their findings was that there is no convincing evidence that Triple P interventions work when applied across the whole population. The authors made this conclusion despite none of the Triple P population studies [8-10] being included in the quantitative meta-analysis. Rather, the strength of evidence from each population study was analyzed according to the authors' own qualitative assessment parameters and included discussion of such issues as randomization, recruitment methods and characteristics of the sample. This subjective and apparently arbitrary assessment of specific aspects of the Triple P population studies is not sufficient grounds to conclude the evidence is not convincing. Wilson *et al. *have overlooked the main contributions of these studies as the forerunners of efforts to build a more comprehensive and inclusive evidence-based population strategy to support all parents. These population-level studies represent a significant advance over approaches that only target a small number of clinic-referred children or parents, by demonstrating the feasibility of reaching broader segments of the parenting population who might benefit from parenting programs, and the potential to prevent morbidity and reduce community prevalence rates.

Wilson *et al. *argue that priority should be given to targeting children with identified problems. This targeting of children whose parents present to services for help or are known to social services creates its own form of sampling bias. Most parents of children with behavior problems (even severe problems), or who maltreat their children, do not seek professional help, and reliance on referral has ensured evidence-based parenting programs are accessible to very few parents [11]. This targeted approach also has greater potential for stigmatizing parents, particularly the most vulnerable, and has done little to address the high prevalence of child behavior problems or child maltreatment despite parenting programs being available for over 30 years in many Western counties [12].

## Other limitations

### Criticisms of recruitment methods

Wilson *et al. *question the value of recruiting parents through media outreach, proposing this method may bias trials because parents are more likely to be motivated than if recruited through other means. Even if this untested assumption were true, parents were randomized to condition and, therefore, motivation is unlikely to account for differential condition effects. Self-referral should not preclude interpretation of intervention outcomes. Many parents have tremendous difficulties accessing parenting services and many report lack of knowledge of available support. It is common practice and a pragmatic necessity in many prevention and treatment trials to employ multiple outreach strategies that include, but are not restricted to, media outreach. This applies to many different types of interventions and not just parenting interventions. Parents are still carefully screened and have to meet inclusion criteria. It should be noted here that for most of the studies cited a range of recruitment strategies were used, and was not solely restricted to media outreach as implied in the review.

### Conclusions relating to paternal data

The authors of the review noted the limited involvement of fathers in Triple P research. However, a lack of father involvement in parenting research is a universal challenge facing all parenting programs - not just Triple P [13]. The authors rightfully point out that there may be alternate explanations for the stronger data derived from maternal reports compared to paternal reports, but these explanations do not translate to the overall conclusions drawn by the authors about father effects.

### No comparison programs

At no stage was Triple P compared to any other parenting or evidence-based psychological intervention with regards to methodological criteria (for example, clinical trial registration, paternal data) to contextualize Triple P's relative performance on these indices.

### Conclusions about observation measures

Wilson *et al. *argue that Triple P has minimal effects on independent observation measures of child outcome. This conclusion is unwarranted primarily because many of the relevant studies demonstrating these effects were excluded. Triple P evolved from a tradition of observational measurement where a series of intra-subject replication experiments [14,15] and a number of group comparison designs [16,17] were used to explore the effects of different treatment components on child and parent outcomes. These early studies all used independent observational measures and demonstrated positive changes on both child and parenting behavior. For prevention and early intervention studies, floor effects create a statistical artefact precluding the demonstration of effects because children's disruptive behaviors on these measures were in the nonclinical range at baseline. In studies where baseline scores are elevated, observational effects are typically noted. Observational methods have serious limitations when problem behaviors are low rate, high intensity, include novel tasks, or create reactivity effects to the presence of observers.

### Criticisms of long-term effects

Wilson *et al. *criticized the use of waitlist control designs as they preclude assessment of long-term effects of intervention. What they fail to point out is that almost all studies included maintenance probes typically showing that post-treatment improvements maintained over various lengths of follow-up, with little evidence of relapse or symptom substitution on child or parent outcomes.

### Clinical trial registration

We agree that clinical trial registration is desirable. Historically, however, trials of psychological, social and educational interventions have typically not been registered on clinical trials databases that were set up largely for drug trials. It is only in recent years that psychological treatment studies have adopted this approach and been required by some journals to be registered on a clinical trials database. Wilson *et al. *do point out that many of the Triple P studies they reviewed took place prior to these guidelines being widely endorsed by mainstream journals or widely disseminated to the field. We now routinely encourage all Triple P trials to be registered. Of note, Wilson *et al. *failed to register the protocol of their own review, despite protocol registration being an important component of the PRISMA statement for reporting systematic reviews and meta-analyses [18].

## Conclusion

When the above concerns are taken together, a picture emerges which shows that Wilson *et al. *report selectively from a limited subsample of available studies, yet make broad ranging conclusions about Triple P that are at variance with the conclusions of other researchers who have examined many of the same studies [19-22]. Further, the impact of parenting programs on other significant family risk and protective factors is ignored. Triple P has a substantial and constantly evolving evidence-base that has accumulated over a 30-year period. This evidence-base includes controlled single case experiments [23]; randomized controlled trials (RCTs) with a variety of comparator conditions, including waitlist controls, care-as-usual controls and active intervention comparisons [24,25]; economic analyses [26-28]; qualitative evaluations of consumer acceptability and cultural relevance [29,30]; quasi-experimental evaluations [9,10]; service-based evaluations [31]; and independent meta-analyses [19-22]. Although not without its limitations, this evidence-base is a testimony to the program developers' sustained commitment to ongoing careful empirical scrutiny of the intervention system, content and processes. Despite this critique, we believe Wilson *et al*., along with the investigators of the studies reviewed, share a united purpose in promoting rigorous scientific evaluation of parenting interventions, and providing timely, quality service provision for families according to their needs.

## Competing interests

The Triple P - Positive Parenting Program is owned by The University of Queensland. The University, through its technology transfer company Uniquest Pty Ltd, has licensed Triple P International Pty. Ltd. to disseminate the program worldwide. Royalties stemming from this dissemination work are paid to UniQuest, which distributes payments to the University of Queensland Faculty of Social and Behavioural Sciences, School of Psychology, Parenting and Family Support Centre, and contributory authors in accordance with the University's intellectual property policy.

No author has any share or ownership in Triple P International. MS is the founder and lead author of the Triple P-Positive Parenting Program, and is a consultant to Triple P International. JP has no competing interests. JK is a co-author of Grandparent Triple P. KT is a co-author of many of the Triple P interventions and resources for families of children up to 12 years of age. AM is a co-author of several Triple P interventions for young children including Fuss-Free Mealtime Triple P. TM is a co-author of Stepping Stones Triple P for families of children with disabilities. AR is a co-author of Teen Triple P for parents of adolescents, and is Head of Training at Triple P International. KS has no competing interests.

## Authors' contributions

All authors assisted with the conceptualization, drafting and editing of the manuscript. All authors read and approved the final manuscript.

## Pre-publication history

The pre-publication history for this paper can be accessed here:

http://www.biomedcentral.com/1741-7015/10/145/prepub

## References

[B1] Wilson P Rush R Hussey S Puckering C Sim F Allely CS Doku P McConnachie A Gillberg C How evidence-based is an 'evidence-based parenting program'? A PRISMA systematic review and meta-analysis of Triple P BMC Med 2012 10 130 10.1186/1741-7015-10-130 23121760 PMC3532197

[B2] Sanders MR Development, evaluation, and multinational dissemination of the Triple P-Positive Parenting Program Annu Rev Clin Psychol 2012 8 345 379 10.1146/annurev-clinpsy-032511-143104 22149480

[B3] Sanders MR The Triple P-Positive parenting program: towards an empirically validated multilevel parenting and family support strategy for the prevention of behavior and emotional problems in children Clin Child Fam Psychol Rev 1999 2 71 90 10.1023/A:1021843613840 11225933

[B4] Sanders MR The Triple P-Positive Parenting Program as a public health approach to strengthening parenting J Fam Psychol 2008 22 506 17 18729665 10.1037/0893-3200.22.3.506

[B5] Olds D In support of disciplined passion J Exp Criminol 2009, 5 201 214

[B6] Nicholson JM Sanders MR Randomized controlled trial of behavioral family intervention for the treatment of child behavior problems in stepfamilies J Divorce Remarriage 1999 30 1 23

[B7] Plant KM Sanders MR Reducing problem behavior during care-giving in families of preschool-aged children with developmental disabilities Res Dev Disabil 2007 28 362 385 10.1016/j.ridd.2006.02.009 16781115

[B8] Prinz RJ Sanders MR Shapiro CJ Whitaker DJ Lutzker JR Population-based prevention of child maltreatment: the U.S. Triple p system population trial Prev Sci 2009 10 1 12 10.1007/s11121-009-0123-3 19160053 PMC4258219

[B9] Sanders MR Ralph A Sofronoff K Gardiner P Thompson R Dwyer S Bidwell K Every family: a population approach to reducing behavioral and emotional problems in children making the transition to school J Prim Prev 2008 29 197 222 10.1007/s10935-008-0139-7 18461457

[B10] Zubrick SR Ward KA Silburn SR Lawrence D Williams AA Blair E Robertson D Sanders MR Prevention of child behavior problems through universal implementation of a group behavioral family intervention Prev Sci 2005 6 287 304 10.1007/s11121-005-0013-2 16160760

[B11] Biglan A Translating what we know about the context of antisocial behavior into a lower prevalence of such behavior J Appl Behav Anal 1995 28 479 492 10.1901/jaba.1995.28-479 8557621 PMC1279854

[B12] Biglan A Flay BR Embry DD Sandler IN The critical role of nurturing environments for promoting human well-being Am Psychol 2012 67 257 271 22583340 10.1037/a0026796 PMC3621015

[B13] Bagner DM Eyberg SM Father involvement in parent training: when does it matter? J Clin Child Adolesc 2003 32 599 605 10.1207/S15374424JCCP3204_13 14710469

[B14] Sanders MR Dadds MR The effects of planned activities and child management procedures in parent training: an analysis of setting generality Behav Ther 1982 13 452 461 10.1016/S0005-7894(82)80007-5

[B15] Sanders MR Glynn EL Training parents in behavioral self-management: an analysis of generalization and maintenance effects J Appl Behav Anal 1981 14 223 237 10.1901/jaba.1981.14-223 7298535 PMC1308209

[B16] Sanders MR McFarland M The treatment of depressed mothers with disruptive children: acontrolled evaluation of cognitive behavioral family intervention Behav Ther 2000 31 89 112 10.1016/S0005-7894(00)80006-4

[B17] Dadds MR Schwartz S Sanders MR Marital discord and treatment outcome in behavioral treatment of childhood conduct disorders J Consult Clin Psychol 1987 55 396 403 3597955 10.1037//0022-006x.55.3.396

[B18] Liberati A Altman DG Tetzlaff J Mulrow C Götzsche PC Ioannidis JPA Clarke M Devereaux P J Kleijnen J Moher D The PRISMA statement for reporting systematic reviews and meta-analyses of studies that evaluate healthcare interventions: explanation and elaboration BMJ 2009 339 b2700 10.1136/bmj.b2700 19622552 PMC2714672

[B19] Nowak C Heinrichs N A comprehensive meta-analysis of Triple P--Positive Parenting Program using hierarchical linear modeling: effectiveness and moderating variables Clin Child Fam Psychol 2008 11 114 144 10.1007/s10567-008-0033-0 18509758

[B20] de Graff I Speetjens P Smit F De Wolff M Tavecchio L Effectiveness of the Triple P Positive Parenting Program on behavioral problems in children: meta-analysis Behav Modif 2008 32 714 735 10.1177/0145445508317134 18475003

[B21] de Graff I Speetjens P Smit F De Wolff M Tavecchio L Effectiveness of the Triple P Positive Parenting Program on parenting: ameta-analysis Fam Relat 2008 57 553 566 10.1111/j.1741-3729.2008.00522.x 18475003

[B22] Thomas R Zimmer-Gembeck MJ Behavioral outcomes of parent-child interaction therapy and Triple P-Positive Parenting Program: areview and meta-analysis J Abnorm Child Psychol 2007 35 475 495 10.1007/s10802-007-9104-9 17333363

[B23] Boyle C Sanders MR Lutzker JR Prinz RJ Shapiro C Whitaker DJ An analysis of training, generalization, and maintenance effects of primary care Triple P for parents of preschool-aged children with disruptive behavior Child Psychiatry Hum Dev 2010 41 114 131 10.1007/s10578-009-0156-7 19697120

[B24] Sanders MR Markie-Dadds C Tully L Bor W The Triple P- Positive Parenting Program: a comparison of enhanced, standard, and self-directed behavioral family intervention for parents of children with early onset conduct problems J Consult Clin Psychol 2000 68 624 640 10965638

[B25] Morawska A Sanders MR An evaluation of a behavioural parenting intervention for parents of gifted children Behav Res Ther 2009 47 463 470 10.1016/j.brat.2009.02.008 19286168

[B26] Mihalopoulos C Sanders MR Turner KMT Murphy-Brennan M Carter R Does the Triple P - Positive Parenting Program provide value for money? Aust NZ J Psychiatry 2008 41 239 246 10.1080/00048670601172723 17464705

[B27] Foster EM Prinz RJ Sanders MR Shapiro CJ The costs of a public health infrastructure for delivering parenting and family support Child Youth Serv Rev 2008 30 493 501 10.1016/j.childyouth.2007.11.002 PMC9380520 35979533

[B28] Aos S Lee S Drake E Pennuci A Klima T Miller M Anderson L Mayfield J Burley M Return on Investment: evidence-based Options to Improve Statewide Outcomes 2011 Document No. 11-07-1201. Olympia, WA: Washington State Institute of Public Policy

[B29] Kirby JN Sanders MR Using consumer input to tailor evidence-based parenting interventions to the needs of grandparents J Child Fam Studies 2012 21 626 636 10.1007/s10826-011-9514-8 PMC3652435 23682208

[B30] Morawska A Sanders MR Goadby E Headley C Hodge L McAuliffe C Pope S Anderson E Is the Triple P-Positive Parenting Program acceptable to parents from culturally diverse backgrounds? J Child Fam Studies 2011 20 614 622 10.1007/s10826-010-9436-x

[B31] Lindsay G Strand S Davis H A comparison of the effectiveness of three parenting programmes in improving parentingskills, parent mental-well being and children's behaviour when implemented on a large scale in community settings in 18 English local authorities: the parenting early intervention pathfinder (PEIP) BMC Public Health 2011 11 962 10.1186/1471-2458-11-962 22208676 PMC3316149

